# Uptake of visibility materials for injury prevention and associated factors among commercial motorcycle riders: a cross-sectional study in Cameroon

**DOI:** 10.1186/s12889-026-28113-6

**Published:** 2026-06-09

**Authors:** Chrisantus Eweh Ukah, Nicholas Tendongfor, Alan Hubbard, Elvis Asangbeng Tanue, Rasheedat Oke, Nahyeni Bassah, Larissa Kumenyuy Yunika, Claudia Ngeha Ngu, S. Ariane Christie, Dickson Shey Nsagha, Alain Chichom-Mefire, Catherine Juillard

**Affiliations:** 1 Department of Public Health and Hygiene, Faculty of Health Sciences, University of Buea, Buea, Cameroon; 2 Division of Epidemiology, School of Public Health, University of California Berkeley, Berkeley, CA USA; 3 Program for the Advancement of Surgical Equity, Department of Surgery, University of California Los Angeles, Los Angeles, CA USA; 4 Department of Surgery and Specialties, Faculty of Health Sciences, University of Buea, Buea, Cameroon

**Keywords:** Visibility materials, Reflective jackets, Knowledge, Attitudes, Practices, Commercial motorcycle riders

## Abstract

**Background:**

Commercial motorcycle riders are among the most vulnerable road users in low- and middle-income countries, with poor visibility contributing significantly to road traffic injuries, particularly under low-light conditions. While visibility materials such as reflective jackets and functional lighting systems are effective, low-cost safety measures, their use remains inconsistent in many settings. In addition to individual behaviors, structural factors such as enforcement, access to safety equipment, and road conditions may influence their uptake. This study assessed the use of visibility materials and factors associated with their uptake among commercial motorcycle riders in the Limbe and Tiko Health Districts of Cameroon.

**Methods:**

A community-based cross-sectional study was conducted among 499 commercial motorcycle riders aged 18 years and above in the Limbe and Tiko Health Districts. Riders were recruited at selected motorcycle pick-up points using a multistage sampling approach. Data were collected using structured interviewer-administered questionnaires and an observational checklist. Descriptive statistics were used to summarize the uptake of visibility materials. Multivariable logistic regression analysis was performed to identify factors independently associated with reflective jacket use, guided by theoretical and contextual relevance. Adjusted odds ratios (AORs) with 95% confidence intervals (CIs) were reported.

**Results:**

The mean age of riders was 32.2 ± 7.6 years, and all participants were male. The most commonly observed visibility feature was a functional headlamp (85.8%), followed by backlights (57.3%) and brake lights (57.3%). Approximately half of the riders were observed wearing reflective jackets (50.9%), while reflective strips were present on only 19.0% of motorcycles. In adjusted analysis, riders who reported obeying traffic regulations were more likely to wear reflective jackets (AOR = 1.52; 95% CI: 1.10–2.30), as were those willing to allocate a budget for visibility materials (AOR = 2.20; 95% CI: 1.30–3.60). Knowledge and attitudes toward visibility materials were also positively associated with reflective jacket use. Each one-point increase in knowledge score was associated with an 18% increase in the odds of reflective jacket use (AOR = 1.18; 95% CI: 1.10–1.26), while each one-point increase in attitude score increased the odds by 14% (AOR = 1.14; 95% CI: 1.07–1.21). Riders reporting safer visibility-related practices were also more likely to wear reflective jackets (AOR = 2.16; 95% CI: 1.36–3.44).

**Conclusion:**

The uptake of visibility materials, particularly reflective jackets and reflective strips, remains suboptimal among commercial motorcycle riders despite widespread availability of basic lighting systems. Both behavioral and contextual factors appear to influence their use. Interventions that combine improved awareness with strategies addressing structural barriers—such as affordability, accessibility, and enforcement—may enhance rider visibility and contribute to reducing road traffic injuries in this population.

**Supplementary Information:**

The online version contains supplementary material available at 10.1186/s12889-026-28113-6.

## Introduction

Road traffic injuries remain a major public health challenge worldwide and are a leading cause of death and disability, particularly among vulnerable road users such as pedestrians, cyclists and motorcyclists [1–4]. Globally, an estimated 1.19 million people die each year as a result of road traffic crashes, with millions more sustaining non-fatal injuries that often result in long-term disability [5]. More than 90% of these deaths occur in low- and middle-income countries, despite these countries having only about 60% of the world’s registered vehicles [6, 7]. Young adults, especially males, are disproportionately affected, resulting in substantial social and economic consequences for families and communities [8, 9].

Road traffic injury prevention is increasingly guided by the Safe System approach, which recognizes that road safety outcomes are shaped not only by individual behavior but also by interactions between road users, vehicles, infrastructure, and policy environments [10]. Within this framework, responsibility for safety is shared across system designers, policymakers, and road users [11]. In many low- and middle-income settings, structural challenges such as poor road infrastructure, inadequate street lighting, weak enforcement of traffic regulations, and limited access to safety equipment may constrain the ability of motorcycle riders to adopt protective behaviors, including the use of visibility materials [11]. Therefore, while individual-level factors remain important, they must be interpreted within the broader system context that influences road user behavior.

Within this broader context, motorcyclists represent one of the most vulnerable groups on the road [12, 13]. In many low- and middle-income countries, motorcycles have become a dominant mode of transport because of their affordability, flexibility, and ability to navigate poor road infrastructure and traffic congestion [14]. While motorcycles play a crucial role in supporting mobility, employment and access to essential services, their increasing use has been accompanied by a marked rise in motorcycle-related crashes and injuries [13]. A substantial proportion of motorcycle collisions involve failures of other road users to detect motorcycles in time, particularly under conditions of poor lighting, high traffic density and complex road environments [15–17].

Limited conspicuity of motorcycles and riders has been consistently identified as an important and preventable contributor to road traffic crashes [17]. Visibility, defined as the ability of a road user to be detected and recognized by others in sufficient time to avoid a collision, is strongly influenced by lighting conditions, environmental contrast, rider positioning and the use of conspicuity-enhancing materials. Visibility materials such as reflective or fluorescent jackets, reflective strips on motorcycles and clothing, functional headlamps, brake lights and trafficator lights substantially increase the detectability of riders, particularly at night, during dawn and dusk, and in adverse weather conditions [18].

Evidence from experimental and observational studies demonstrates that high-visibility clothing and reflective materials significantly improve the visual detection and recognition distance of motorcyclists by other road users [18]. Reflective and fluorescent materials enhance contrast against complex backgrounds and extend the time available for drivers to respond appropriately. In settings where motorcycles share congested road space with multiple vehicle types, pedestrians and informal roadside activities, the potential safety benefit of improved conspicuity becomes even more critical [19].

Although visibility materials have been shown to improve rider conspicuity, their uptake and consistent use among commercial motorcycle riders remain variable across low- and middle-income countries [20]. Barriers such as limited awareness of their protective role, cost, discomfort, aesthetic preferences, inadequate enforcement of road safety regulations, and low risk perception contribute to poor adoption. In addition, road safety interventions in many African settings have traditionally focused on helmets and licensing, while conspicuity measures have received comparatively less attention within policy, enforcement, and health promotion programs.

Sub-Saharan Africa has the highest road traffic injury mortality rate globally. Motorcycle crashes account for a growing proportion of injuries and deaths in both urban and peri-urban areas, where commercial motorcycle transport has rapidly expanded [21]. The increasing reliance on motorcycles as a primary source of income for young people, combined with weak regulatory environments and limited infrastructure for non-motorized and informal transport, has intensified the vulnerability of riders and their passengers [22].

In Cameroon, road traffic injuries remain a major public health concern, with an estimated road traffic mortality rate of approximately 24 deaths per 100,000 population, according to the World Health Organization Global Status Report on Road Safety [23]. Road transport is the predominant means of mobility, and commercial motorcycle transport—commonly known as “okada”—has become an essential component of urban and peri-urban transport systems [22]. This is particularly evident in the South-West Region, where difficult terrain, limited public transport coverage and socio-economic disruptions have increased reliance on motorcycles for commuting, trade and access to health and social services. However, this expansion has been accompanied by persistently high levels of motorcycle-related crashes and injuries. In Cameroon, road traffic injuries remain a leading cause of morbidity and mortality, with motorcycle-related crashes accounting for a substantial and increasing proportion of cases reported in urban health facilities.

Limbe and Tiko Health Districts are among the most active commercial motorcycle transport hubs in the South-West Region of Cameroon. These districts experience heavy traffic flow, mixed road use by vehicles and pedestrians, limited street lighting in many areas and frequent operation of motorcycles during early morning and late evening hours. Such environmental and infrastructural conditions create a high-risk context in which poor rider visibility substantially increases the likelihood of collisions. The ongoing socio-political crisis in the region has further altered mobility patterns, increased population movements and placed additional strain on already limited road safety and health system capacities.

Although visibility materials such as reflective jackets, reflective strips and functional lighting systems are recognized as essential components of motorcycle safety, empirical evidence on their use and determinants among commercial motorcycle riders in Cameroon remains limited. Existing research in the country has largely focused on personal protective equipment such as helmets and protective clothing, with comparatively little attention given to conspicuity-related measures. Consequently, there is insufficient context-specific evidence to inform targeted interventions aimed at improving the visibility of riders and motorcycles in high-risk settings.

Understanding the factors influencing the adoption of visibility materials among commercial motorcycle riders is critical for designing effective and context-specific road safety interventions.

This study therefore aimed to assess the knowledge, attitudes, and use of visibility materials and to identify factors associated with their uptake among commercial motorcycle riders in the Limbe and Tiko Health Districts of Cameroon. Generating this evidence is essential for strengthening motorcycle safety programming, informing public health and transport policy, and contributing to the reduction of road traffic injuries in a region where motorcycles remain a vital but high-risk mode of transport.

## Materials and methods

### Study design and setting

A community-based cross-sectional study was conducted among commercial motorcycle riders in the Limbe and Tiko Health Districts of the South-West Region of Cameroon from May 15 to August 17, 2024. These districts are major urban and peri-urban transport hubs characterized by a high reliance on commercial motorcycle transport due to traffic congestion, limited public transportation, and challenging road infrastructure. The study area is also affected by ongoing sociopolitical instability, which has increased dependence on motorcycles for daily mobility and income generation.

### Study population and eligibility criteria

The study population consisted of commercial motorcycle riders aged 18 years and above who were actively operating within the Limbe and Tiko Health Districts during the study period.

#### Inclusion criteria

Riders aged ≥ 18 years, who had been operating in the study area for at least six months prior to the survey, and who provided informed consent.

#### Exclusion criteria

Riders who were temporarily unable to participate due to illness or other health-related conditions at the time of data collection.

### Sample size determination

The sample size was calculated using Cochran’s formula for estimating proportions in cross-sectional studies. A 95% confidence level, a margin of error of 5%, and an assumed prevalence of 50% were used due to the absence of prior estimates on the uptake of visibility materials in the study setting.

The sample size was adjusted using a design effect of 1.3 to account for clustering at motorcycle pick-up points, assuming moderate intra-cluster correlation. A 5% allowance for non-response was also included. The final sample size was 499 commercial motorcycle riders.

### Sampling procedure

A multistage sampling approach was employed. First, Limbe and Tiko Health Districts were purposively selected due to their high concentration of commercial motorcycle riders and intense traffic activity. A sampling frame of 132 major motorcycle pick-up points (98 in Limbe and 34 in Tiko) was developed with the support of local motorcycle unions and transport authorities.

In the second stage, 80% of the pick-up points were randomly selected, yielding 105 clusters (78 in Limbe and 27 in Tiko). Within each selected cluster, riders were recruited consecutively as they arrived at or departed from the pick-up points during data collection periods. Data collection was conducted during both weekdays and weekends and included morning and afternoon periods to enhance representativeness. The final sample included 300 riders from Limbe and 199 from Tiko, proportionate to the estimated distribution of riders across the two districts.

A total of 588 commercial motorcycle riders were approached across the selected pick-up points during the study period, of whom 499 consented to participate, yielding a response rate of 84.9%. Non-participation was primarily due to time constraints and lack of interest.

### Data collection tools and procedures

Data were collected using a structured interviewer-administered questionnaire and an observational checklist.

The questionnaire captured information on socio-demographic characteristics, riding experience, history of road traffic crashes, compliance with traffic regulations, knowledge and attitudes regarding visibility materials, and other road safety-related behaviors.

The observational checklist was used to document the presence and use of visibility materials, including reflective jackets or vests, reflective strips or stickers, and functional lighting systems such as headlamps, brake lights, rear lights, and trafficator lights.

The tools were adapted from previously published studies on road safety behaviors and were reviewed for content validity by three public health experts. They were pretested among 30 commercial motorcycle riders in Buea, a district with similar characteristics but not included in the study. Necessary revisions were made based on pretest findings.

Internal consistency of the knowledge and attitude items was assessed using Cronbach’s alpha, yielding a reliability coefficient of 0.78.

Data were collected electronically using the Kobo Collect platform on Android devices, which enabled real-time data capture, skip logic, and secure storage. Eight trained research assistants conducted the interviews after receiving two days of training on study procedures, ethical considerations, and data collection techniques. Interviews were conducted primarily in English, with clarification provided in Pidgin English where necessary.

### Measurement of study variables

#### Outcome variable

The primary outcome was the use of reflective jackets, assessed through direct observation at the time of the survey and confirmed by participant report. Riders observed wearing reflective jackets during routine operation were classified as users, while others were classified as non-users. In this study, reflective jacket use was considered a proxy measure of visibility-related practice, as it represents a key observable behaviour related to rider conspicuity.

#### Explanatory variables

Explanatory variables included socio-demographic characteristics, riding experience, compliance with traffic regulations, willingness to invest in safety equipment, knowledge, attitudes, and visibility-related practices.

Knowledge of visibility materials was assessed using six structured questions, with each correct response scored as one point, resulting in a total score range of 0 to 8.

Attitudes toward visibility materials were assessed using eight Likert-scale items measuring perceptions of usefulness, comfort, and importance. Responses ranged from strongly disagree to strongly agree, yielding a total score of between 8 and 40.

Knowledge and attitude scores were treated as continuous variables in regression analyses to preserve statistical information.

### Data management and analysis

Data were exported from Kobo Collect into SPSS version 26 for cleaning and analysis. Data quality checks included assessment of completeness, consistency, and outliers.

Descriptive statistics were used to summarize participant characteristics and visibility material use. Categorical variables were presented as frequencies and percentages, while continuous variables were summarized using means and standard deviations.

Logistic regression analysis was performed to examine factors associated with reflective jacket use. Selection of variables for the multivariable model was guided by theoretical relevance and evidence from prior literature, rather than solely on statistical significance.

Multivariable logistic regression was used to identify factors independently associated with reflective jacket use while controlling for potential confounders. Adjusted odds ratios (AORs) with 95% confidence intervals were reported, and statistical significance was set at *p* < 0.05.

## Results

### Socio-demographic and riding characteristics of respondents

A total of 499 commercial motorcycle riders participated in the study. The mean age of the riders was 32.2 ± 7.6 years. Most riders were aged 21–30 years (48.5%), followed by those aged 31–40 years (38.5%). All participants were male.

More than half of the riders were single (52.3%), and the majority had attained secondary level education (58.3%). A substantial proportion of respondents reported having previously been involved in a road traffic crash (68.3%). A total of 352 (70.5%) of respondents were observed not wearing helmets while riding.

Most riders reported that they obey traffic regulations while riding (75.2%), and 89.6% indicated willingness to allocate money to purchase visibility materials (Table 1). These self-reported behaviors should be interpreted with caution due to the potential for social desirability bias.

**Table 1:** Socio-demographic and riding characteristics of respondents

Variable	Category	Frequency	Percentage
Age group (years)	21–30	242	48.5
	31–40	192	38.5
	41–50	57	11.4
	50+	8	1.6
	Total	499	100
Sex	Male	499	100
	Total	499	100
Marital status	Single	261	52.3
	Married	228	45.7
	Divorced	10	2
	Total	499	100
Highest level of education	No formal	19	3.8
	Primary	149	29.9
	Secondary	291	58.3
	Tertiary	40	8
	Total	499	100
Have been involved in a road traffic crash	No	158	31.7
	Yes	341	68.3
	Total	499	100
Obey traffic regulations when riding	No	124	24.8
	Yes	375	75.2
	Total	499	100
Will to allocate money to buy VM	No	52	10.4
	Yes	447	89.6
	Total	499	100
Rider observed wearing helmet while riding	Yes	147	29.5
	No	352	70.5
	Total	499	100

### Uptake of visibility materials among commercial motorcycle riders

**Fig. 1:**
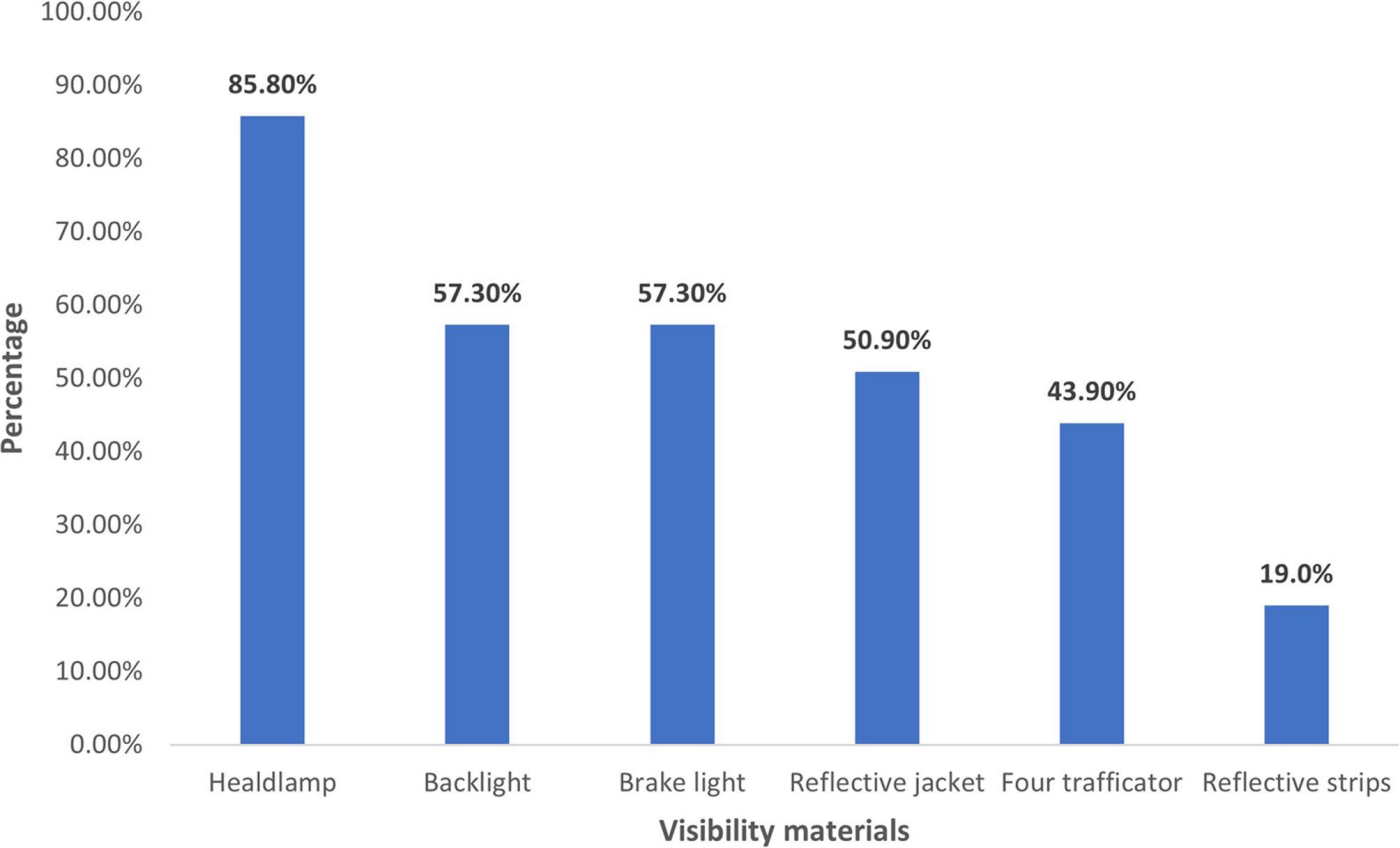
Visibility materials use by commercial motorcycle riders

The uptake of visibility materials varied across different components (Fig. 1). The most commonly observed feature was a functional headlamp (85.8%), followed by functional backlights (57.3%) and brake lights (57.3%).

Approximately half of the riders were observed wearing reflective jackets (50.9%), which constituted the primary outcome of this study. In addition, 43.9% of motorcycles had four functioning trafficator lights, while reflective strips were present on only 19.0% of motorcycles, making them the least commonly observed visibility material.

Overall, while built-in lighting systems were relatively common, the use of additional visibility-enhancing materials such as reflective jackets and reflective strips was comparatively low.

### Knowledge of motorcycle riders regarding visibility materials

Overall, knowledge of visibility materials varied among respondents (Table 2). A majority of riders (61.1%) correctly identified the primary purpose of visibility materials as improving visibility to other road users. However, only 45.3% reported that visibility materials should be used at all times while riding. Less than half of the riders correctly identified yellow as the most visible color during the day (47.3%) or recognized that visibility materials should be placed on both the front and back of the motorcycle (49.7%). Regarding maintenance practices, 47.3% reported that visibility materials should only be inspected when a problem is noticed, indicating gaps in preventive safety practices.

**Table 2:** Knowledge of commercial motorcycle riders on visibility materials

Variable	Category	Frequency	Percentage
Primary purpose of visibility material	I do not know	61	12.2
	To beautify the motorcycle	110	22.0
	Improve visibility to road users	305	61.1
	To reduce vehicle weight	23	4.6
	Total	499	100
When to use visibility material	At all times when riding	226	45.3
	Only at night	173	34.7
	Only during raining weather	66	13.2
	Only during the day	34	6.8
	Total	499	100
Most visible colour during the day	Green	142	28.5
	Red	98	19.6
	White	23	4.6
	Yellow	236	47.3
	Total	499	100
Recommended placement of visibility materials	I do not know	52	10.4
	Both front and back of the bike	248	49.7
	Only on the back of the bike	142	28.5
	Only on the front of the bike	57	11.4
	Total	499	100
Frequency of inspection of visibility materials	Before every ride	127	25.5
	I do not know	47	9.4
	Once a year	89	17.8
	Only when they notice a problem	236	47.3
	Total	499	100

**Fig. 2:**
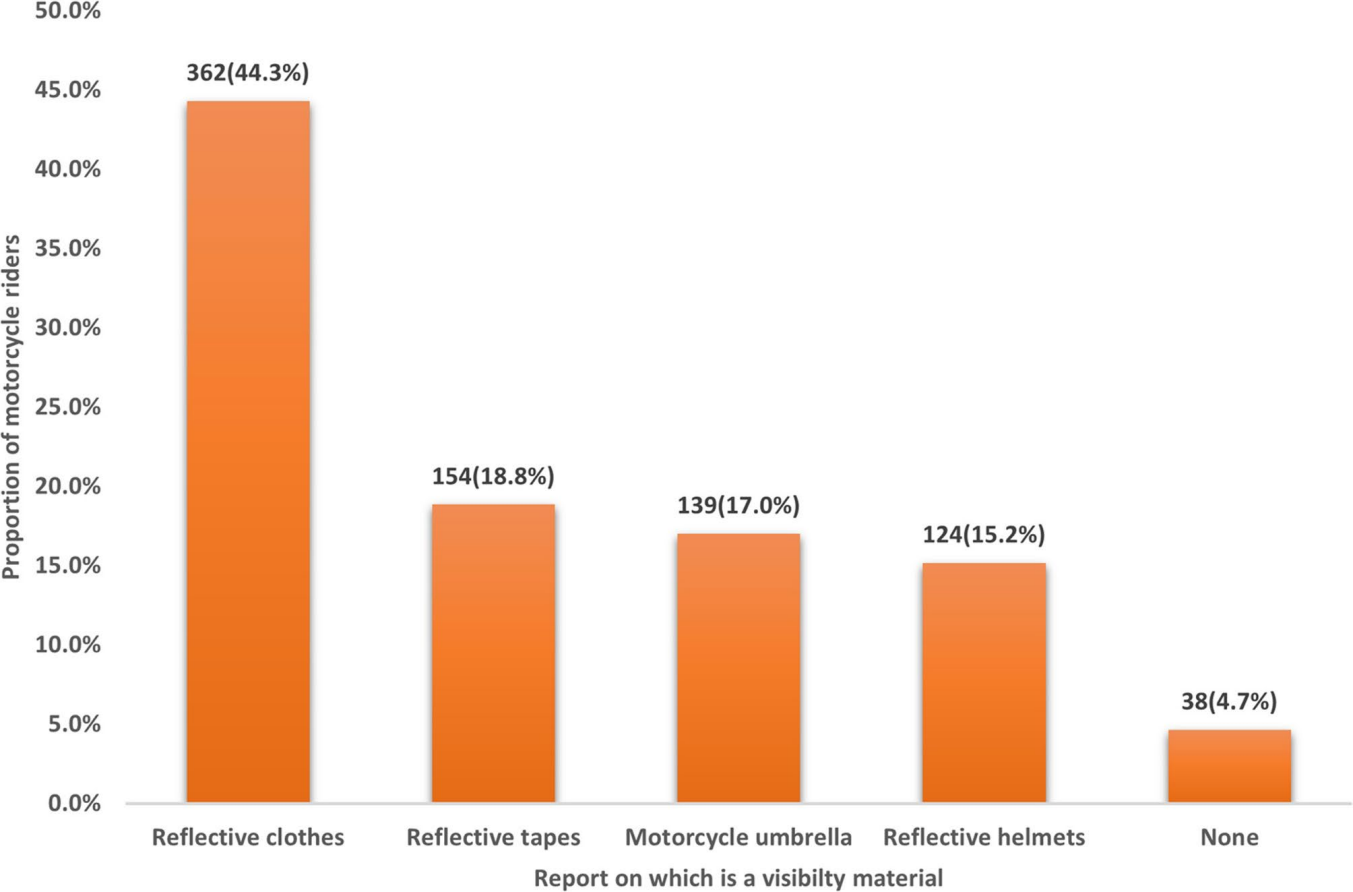
Distribution of participants according to their report of which is a visibility material

When asked to identify visibility materials (Fig. 2), reflective clothing was the most frequently reported item.

### Attitudes of motorcycle riders toward visibility materials

With respect to the attitudes of commercial motorcycle riders on the use of visibility materials when riding (Table 3), 316 (63.3%) agreed that reflective materials are important to commercial motorcycle when riding and 280 (56.1%) agreed that reflective materials enhance visibility to other road users. A total of 294 (58.9%) agreed that they were comfortable using visibility materials and 234 (46.9%) agreed that using reflective stickers on the motorcycle was important. About half of the riders 250 (50.1%) agreed that they would use visibility materials even if usage is not a legal requirement and 263 (52.7%) agreed that the use of visibility materials was necessary for all commercial motorcycle riders.

**Table 3:** Attitudes of commercial motorcycle riders towards visibility materials

Variable	Strongly disagree	Disagree	Neutral	Agree	Strongly agree
Reflective materials are important	0(0)	27(5.4)	61(12.2)	316(63.3)	95(19.0)
Reflective tapes enhance visibility on the road	0(0)	50(10)	90(18.0)	280(56.1)	79(15.8)
Comfortable using reflective clothing while riding	2(0.4)	51(10.2)	85(17.0)	294(58.9)	67(13.4)
It is important to use reflective stickers	8(1.6)	73(14.6)	119(23.8)	234(46.9)	65(13.0)
will use VM even if it is not a legal requirement	6(1.2)	74(14.8)	97(19.4)	250(50.1)	72(14.4)
The use of visibility materials is a necessity	2(0.4)	64(12.8)	81(16.2)	263(52.7)	89(17.8)
feel comfortable with enforcement of using VM	7(1.4)	63(12.6)	105(21.0)	258(51.7)	66(13.2)
Perceive overall use of VM as very important	39(7.8)	87(17.4)	281(56.3)	90(18.0)	2(0.4)

### Factors associated with reflective jacket use among commercial motorcycle riders

In bivariable analysis, several factors were associated with reflective jacket use, including compliance with traffic regulations, willingness to allocate a budget for visibility materials, knowledge score, attitude score, and visibility-related practices.

In the multivariable logistic regression model, selected based on theoretical relevance and prior evidence, six variables remained independently associated with reflective jacket use (Table 4).

**Table 4:** Factors associated with reflective jacket use among commercial motorcycle riders

Variable	Category	COR	AOR	95% CI (AOR)	*p*-value
Age group	21–30	1	1	Reference	
	31–40	0.89	0.95	0.62–1.45	0.81
	41–50	0.8	0.9	0.50–1.60	0.72
	> 50	0.53	0.7	0.20–2.10	0.51
Marital status	Single	1	1	Reference	
	Married	0.82	0.9	0.63–1.30	0.58
Education	No formal	1	1	Reference	
	Primary	0.92	0.95	0.40–2.20	0.91
	Secondary	1.10	1.05	0.50–2.40	0.88
	Tertiary	1.25	1.10	0.45–2.80	0.82
Previous crash involvement	No	1	1	Reference	
	Yes	1.13	1.2	0.80–1.80	0.36
Obeys traffic regulations	No	1	1	Reference	
	Yes	1.77	1.52	1.10–2.30	0.046
Willing to allocate budget for visibility materials	No	1	1	Reference	
	Yes	2.45	2.2	1.30–3.60	0.002
Knowledge score (per unit increase)	Continuous	1.34	1.18	1.10–1.26	0.001
Attitude score (per unit increase)	Continuous	1.16	1.14	1.07–1.21	0.003
Rider wearing a helmet	No	1	1	Reference	
	Yes	3.43	3.49	1.98–6.12	<0.001

Riders who reported obeying traffic regulations had higher odds of wearing reflective jackets compared with those who did not (AOR = 1.52; 95% CI: 1.10–2.30). Similarly, riders willing to allocate a budget for visibility materials were more than twice as likely to wear reflective jackets (AOR = 2.20; 95% CI: 1.30–3.60). Helmet use was also independently associated with reflective jacket use. Riders who were observed wearing helmets had significantly higher odds of wearing reflective jackets compared with non-helmet users (AOR = 3.49; 95% CI: 1.98–6.12). This finding suggests that the uptake of visibility materials may occur alongside other protective riding behaviours among commercial motorcycle riders.

Knowledge and attitudes toward visibility materials were also significantly associated with reflective jacket use. Each one-point increase in knowledge score was associated with an 18% increase in the odds of reflective jacket use (AOR = 1.18; 95% CI: 1.10–1.26), while each one-point increase in attitude score increased the odds by 14% (AOR = 1.14; 95% CI: 1.07–1.21).

Age group, marital status, education, and previous involvement in a road traffic crash were not significantly associated with reflective jacket use after adjustment for potential confounders.

## Discussion

This study assessed the uptake of visibility materials and factors associated with their use among commercial motorcycle riders in the Limbe and Tiko Health Districts of Cameroon. The findings indicate that although most riders possessed functional lighting systems, the uptake of additional conspicuity-enhancing materials, particularly reflective jackets and reflective strips, remained suboptimal. In addition, behavioural and cognitive factors such as compliance with traffic regulations, willingness to invest in safety equipment, knowledge of visibility materials, and attitudes toward their use were independently associated with reflective jacket use.

The findings show that basic lighting systems integrated into motorcycles were widely present among riders, with the majority having functional headlamps and more than half having functioning backlights and brake lights. However, only about half of the riders were observed wearing reflective jackets and fewer than one in five motorcycles had reflective strips. These results suggest that riders may be more likely to maintain visibility features that are mechanically integrated into motorcycles than those requiring personal initiative or additional financial investment. Similar patterns have been reported in other low- and middle-income settings, where the uptake of conspicuity-enhancing materials such as reflective clothing remains lower than that of essential lighting equipment [24].

The low use of reflective strips observed in this study is concerning, particularly in settings characterized by limited street lighting and complex traffic interactions such as those in Limbe and Tiko. In such environments, inadequate rider visibility may increase the likelihood of delayed detection by other road users, thereby elevating crash risk [25]. These findings highlight the need for interventions that promote the consistent use of both wearable and vehicle-based visibility materials.

The study also revealed variability in riders’ knowledge regarding the appropriate use and maintenance of visibility materials. While a majority of respondents correctly identified the role of visibility materials in improving visibility, knowledge about optimal usage conditions and routine inspection practices was less consistent. Previous research has shown that knowledge of road safety measures can influence protective behaviours among motorcyclists. However, knowledge alone may be insufficient to ensure consistent behavioural adoption, particularly in the presence of structural and contextual barriers.

Attitudes toward visibility materials were generally positive but not uniformly so. While many respondents acknowledged the importance of reflective materials for safety, a substantial proportion expressed neutral views regarding their consistent use. Attitudinal factors have been widely recognized as important determinants of safety-related behaviours, particularly in transport settings where compliance depends on perceived risk, benefit, and convenience [26]. Negative perceptions related to comfort, aesthetics, or inconvenience may limit the routine use of reflective clothing even among riders who are aware of its benefits.

The multivariable analysis showed that riders who reported obeying traffic regulations were more likely to wear reflective jackets. This finding suggests that the use of visibility materials may be part of a broader pattern of safety-oriented behaviour. Riders who adhere to traffic regulations may also be more receptive to safety messages and more likely to adopt additional protective practices. Similar clustering of safety behaviours has been reported in previous studies [27].

Willingness to allocate financial resources for visibility materials was also strongly associated with reflective jacket use. Riders who reported willingness to invest in safety equipment were more than twice as likely to wear reflective jackets. This finding suggests that financial considerations may influence the uptake of visibility materials. In many low- and middle-income settings, commercial motorcycle transport operates within informal economic systems where income is often unstable, and safety investments may compete with other priorities [1].

The study further found that riders who wore helmets were significantly more likely to wear reflective jackets. This finding supports the notion that the use of visibility materials may form part of a broader cluster of safety-oriented riding behaviours among commercial motorcycle riders. Similar behavioural clustering has been observed in previous road safety studies, where individuals who adopt one protective behaviour are more likely to engage in additional safety practices [1]. Helmet use may therefore reflect greater risk awareness, stronger safety attitudes, or increased compliance with road safety recommendations among riders.

Knowledge and attitudes toward visibility materials were also independently associated with reflective jacket use. Riders with higher knowledge scores and more favourable attitudes were more likely to use reflective jackets. These findings are consistent with behavioural frameworks such as the Health Belief Model, which posit that individuals are more likely to adopt preventive behaviours when they perceive benefits and fewer barriers to action [26].

Interestingly, previous involvement in a road traffic crash was not significantly associated with reflective jacket use after adjustment. This suggests that prior exposure to road traffic injury does not necessarily translate into improved safety behaviour. Similar findings have been reported in other studies where experience of crashes did not consistently lead to sustained adoption of protective measures. This may reflect normalization of risk or fatalistic attitudes among riders operating in high-risk environments.

Importantly, these findings should be interpreted within a broader systems perspective. While this study focused on individual-level determinants, structural factors such as road infrastructure, availability and affordability of visibility materials, and enforcement of road safety regulations likely play a critical role in shaping rider behaviour. This aligns with the Safe System approach, which emphasizes that road safety outcomes result from interactions between individuals and the wider transport system, and that responsibility for safety should not be placed solely on road users.

The findings of this study have implications for road safety interventions targeting commercial motorcycle riders. Interventions should not focus solely on increasing awareness but should also address barriers related to access, affordability, and consistent use of visibility materials. Strategies such as subsidized provision of reflective jackets, integration of visibility requirements into enforcement frameworks, and collaboration with motorcycle unions and transport associations may help improve uptake. Such approaches should be implemented alongside broader system-level improvements, including infrastructure, lighting, and regulatory enforcement.

### Strengths and limitations

A major strength of this study is the relatively large sample size and the use of both direct observation and interviewer-administered questionnaires, which reduced reliance on self-report alone. Furthermore, the study was conducted in a crisis-affected setting, providing valuable evidence from an under-studied population of high-risk road users.

However, several limitations should be considered. The cross-sectional design limits the ability to establish causal relationships between the identified factors and visibility material use. In addition, some variables, including compliance with traffic regulations and willingness to invest in safety equipment, were self-reported and may be subject to social desirability bias. The study also did not directly assess actual behavioural practices over time, which limits the ability to fully capture consistent use patterns of visibility materials. Although reflective jacket use was used as a proxy for visibility-related practice, the study did not comprehensively assess other dimensions of practice such as frequency, consistency of use, or context-specific behaviours. This may limit the ability to fully capture the range of visibility-related practices among riders. Despite these limitations, the study provides important baseline evidence for designing targeted interventions and informing future research.

## Conclusion

The uptake of visibility materials among commercial motorcycle riders in the Limbe and Tiko Health Districts remains suboptimal, particularly for reflective jackets and reflective strips, despite generally good knowledge and widespread availability of basic lighting systems. Use of visibility materials was independently associated with knowledge, attitudes, compliance with traffic regulations, helmet use, and willingness to invest in safety equipment. The observed association between helmet use and reflective jacket use suggests that visibility material uptake may form part of a broader pattern of safety-oriented riding behaviours among commercial motorcycle riders. These findings suggest that awareness alone is insufficient to improve safety behaviour. Interventions that combine behavioural approaches with strategies addressing structural and economic barriers are needed to enhance rider visibility and reduce the risk of road traffic injuries among commercial motorcycle riders.

## Supplementary Information

Supplementary Material 1.

## Data Availability

The datasets generated and/or analysed during the current study are available in the supplementary files submitted alongside the manuscript. The dataset contains no personal identifiers or confidential participant information.

## Abbreviations

AOR: Adjusted Odds Ratio
CI: Confidence Interval
IRB: Institutional Review Board
LMICs: Low- and Middle-Income Countries
RTIs: Road Traffic Injuries
SPSS: Statistical Package for the Social Sciences
WHO: World Health Organization
